# Reconstruction for diverse fronto-orbital defects with computer-assisted designed and computer-assisted manufactured PEEK implants in one-stage operation: Case reports

**DOI:** 10.1097/MD.0000000000027452

**Published:** 2021-10-08

**Authors:** Min Yang, Zhangyi Wu, Hai Yu, Jun Cheng

**Affiliations:** a Department of Ophthalmology, Tongde Hospital of Zhejiang Province, Hangzhou, China; b Department of Neurosurgery, The Affiliated Hospital of Hangzhou Normal University, Hangzhou, China; c Department of Neurosurgery, Tongde Hospital of Zhejiang Province, Hangzhou, China.

**Keywords:** computer-assisted designed and computer-assisted manufactured, fronto-orbital defect, one-stage operation, polyetheretherketone implant, reconstruction

## Abstract

**Rational:**

Reconstruction of complex craniofacial defects in fronto-orbital region has been reported to be extremely few. In this study, we report 2 cases with fronto-orbital defects of different etiologies in one-stage surgical reconstruction with polyetheretherketone (PEEK) prosthesis using computer-assisted design and computer-assisted manufactured (CAD–CAM) techniques.

**Patient concerns:**

One patient was a 49-year-old man, who admitted with a depressed and comminuted fracture in the left fronto-orbital region as a result of a motor vehicle collision. The other patient was a 45-year-old woman who was hospitalized with an unexpected diagnosis of a fronto-orbital bone tumor during a head CT examination in a minor traumatic brain injury. None of them had a significant past medical history.

**Diagnoses:**

The first patient's head computed tomography (CT) showed multiple depressed comminuted fractures in the right fronto-orbital region with localized frontal lobe contusion, and the diagnosis was clear when combined with the mechanism of traumatic head injuries. The second patient's head CT and magnetic resonance image suggested a right lateral orbital neoplastic lesion that distorted peripheral bone, the postoperative pathological examination demonstrated an osteoma with fibromatous hyperplasia, and thus the women's diagnosis was confirmed.

**Interventions:**

A three-dimensional image of both patients’ skull bone were collected from a high-resolution CT. A virtual surgical planning for lesion excision and defect remodeling based on CAD–CAM techniques was undertaken, and than the reconstruction surgery was performed in a single procedure using PEEK prosthesis. Antibacterial treatment was prescribed routinely.

**Outcomes:**

Postoperatively, both patients achieved excellent aesthetic restoration as well as functional recovery of the orbital cavity without neurological or infectious complications during an average 22 months follow-up.

**Lessons:**

The CAD–CAM PEEK implants could be a preferred option for reconstruction of patients with various complex fronto-orbital defects.

## 1 Introduction

Cranial defects in the fronto-orbital region may be due to a variety of different pathologies, such as intracranial/extracranial infections, tumor surgery, or cranio-facial trauma settings.^[1–3]^ Traditional materials and techniques commonly used for fronto-orbital reconstruction include autologous bone grafts, titanium mesh (TM), polymethyl methacrylate, and hydroxyapatite in primary or second operation.^[1,2,4,5]^ Each material certainly has its advantages and yet, inevitably they are restricted to some distinct disadvantages, such as unpredictable resorption rates of autologous bone grafts, lack of contourability, poor plasticity, incompatibility, complex installation, and incorrect implant placement.^[1–7]^

In the past decade, computer-assisted designed and computer-assisted manufactured (CAD–CAM) prostheses have been increasingly used in the medical field with the research and development of new biomaterials and computational three-dimensional (3D) rapid prototyping technologies.^[1,3,8]^ However, this technique of using polyetheretherketone (PEEK) to reconstruct fronto-orbital bone defects has been rarely reported. In this study, we collected the clinical characteristics, CAD–CAM PEEK implantation procedure and outcomes of 2 cases with diverse fronto-orbital bone defects, so as to improve the understanding of this sub-type of individuals. This study was approved by the Clinical Research and Ethics Committee at the Affiliated Hospital of Hangzhou Normal University, and written consents were obtained from the patients for publication.

## 2 Case report

### 2.1 Clinical presentation

#### 2.1.1 Case one

**Figure 1:**
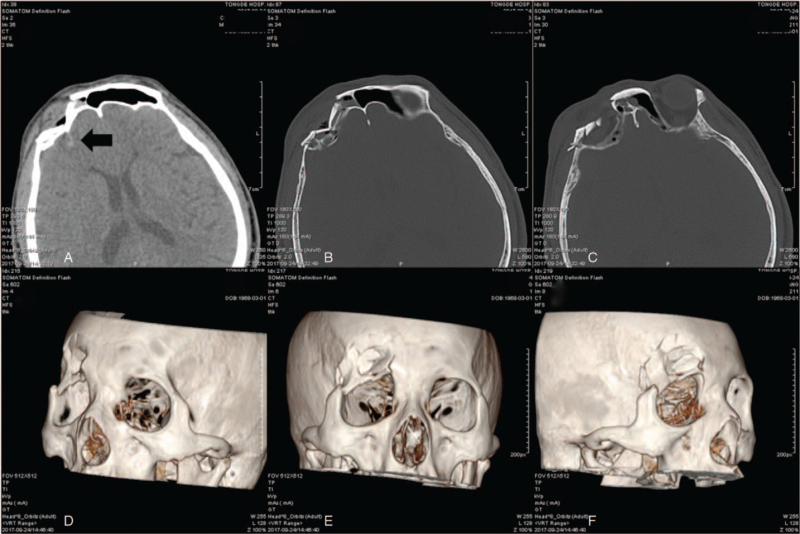
Complex traumatic fracture of right fronto-orbital, lateral orbital, and orbital roof regions in case 1: axial and fronto-orbital high-resolution computer tomography scan (A–C, the arrow showed localized frontal lobe contusion) and three-dimensional reconstruction of the skull fracture (D–F).

A 49-year-old man suffered severe anterior cranial and periorbital injuries with transient loss of consciousness in an motorcycle accident, and a computed tomography (CT) scan on admission revealed multiple depressed comminuted fractures in the right frontal, lateral orbital, and orbital roof regions, as well as localized frontal lobe contusions (Fig. 1). No obvious neurological defects were found preoperatively, and the eye examination of visual acuity and visual field, pupillary reflex and funduscopy were normal, but extraocular movements were found to be limited when the left superior rectus muscle looked upward. The patient has no significant past medical history. A preoperative 3D rapid prototype reconstruction model was established to capture the entire cranio-facial skeleton and defect according to CAD–CAM technique based on high-resolution CT scan (with slice thickness of 1 mm).

#### 2.1.2 Case two

**Figure 2:**
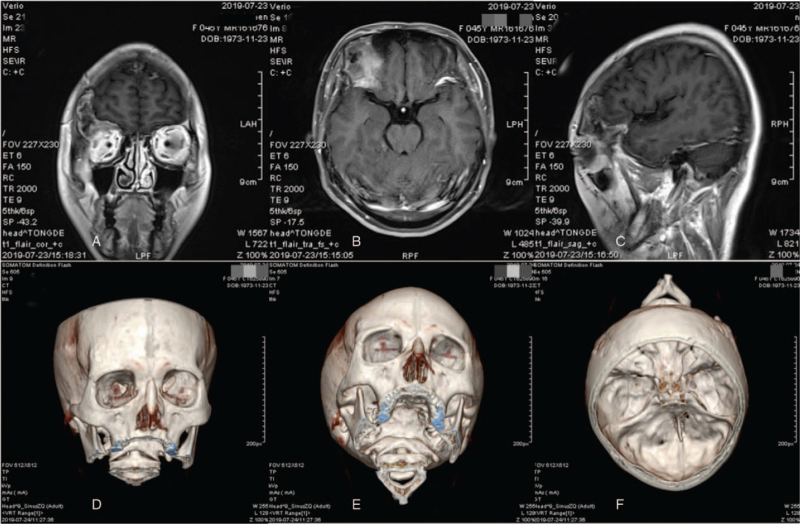
Neoplastic lesion in the right frontal/supraorbital and anterior skull base regions in case 2: different phases of enhancing magnetic resonance imaging (A–C) and three-dimensional reconstruction of the skull tumor by high-resolution computer tomography (D–F).

A 45-year-old lady required a head CT examination for a mild head collision, which showed no positive evidence of trauma but unexpectedly suggested a right lateral orbital osteoma. The patient did not complain of any neurological or orbital disorder and had no significant past medical history. Further head-enhanced magnetic resonance image confirmed a large (>4 cm) fronto-orbital neoplastic lesion that distorted the right frontal bone and the orbital roof (Fig. 2). In addition, a high-resolution CT scan of the entire skull was performed using CAD–CAM technology, which was used to surgically simulate the extent of tumor resection, to determine the borders of the bone defect, and to design and fabricate an implant prosthesis in an appropriate program.

### 2.2 Implant design and manufacture

**Figure 3:**
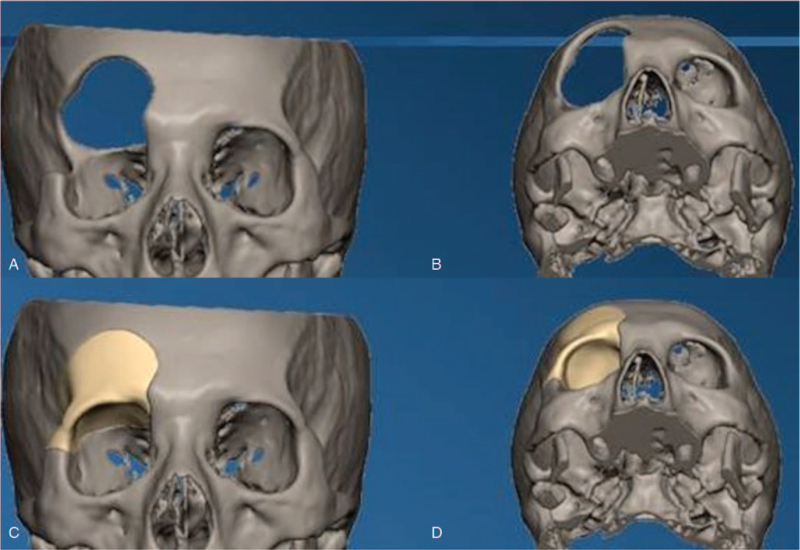
Virtual surgical planning of cranioplasty by computer-assisted design and computer-assisted manufacturing techniques to determine the extent of defects and the process fabrication of PEEK implants in case 1.

**Figure 4:**
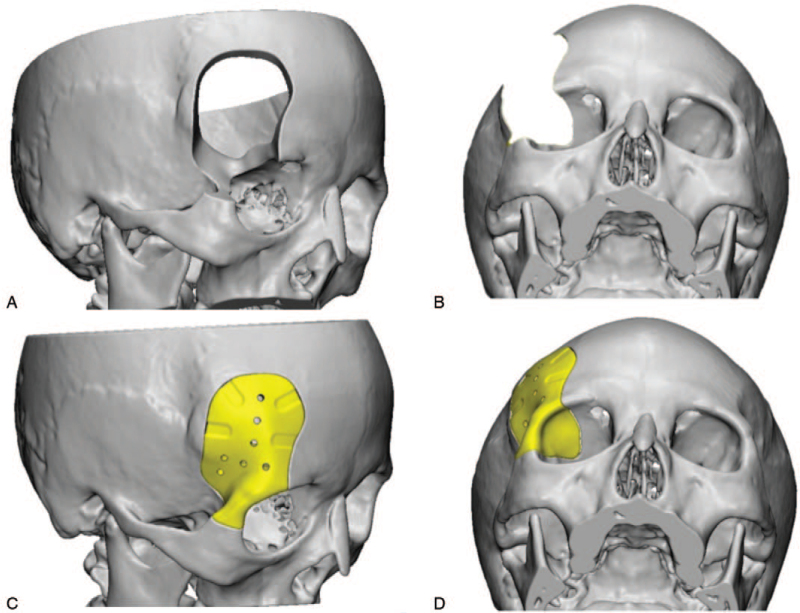
Virtual surgical planning of cranioplasty by computer-assisted design and computer-assisted manufacturing techniques to determine the extent of defects and the process fabrication of PEEK implants in case 2.

The 3D high-resolution CT data was transferred to a data storage device in DICOM format and forward to a medical manufacturing company (L1Wn-S3T2, Shenzhen, China) to generate the respective digital 3D models using FreeForm Modeling Plus software (Version 10.0) (Shenzhen, China). The contour and curvature of the prosthetic models were derived from the natural stereo-skeleton of the patient's normal side of the skull, constructing a mirror image of the external cranial surface that can perfectly match the defect on the other side. Once the virtual surgical planning was completed, the range of craniectomy and skull defect boundary were determined. These patient-specific surgical guides and custom-made implants were produced using CAD–CAM 3D rapid prototyping technology after final confirmation was completed by the surgeon and technical engineer (Figs. 3 and 4). The prefabricated PEEK implants were delivered and sterilized by heat in our hospital disinfection room.

### 2.3 Surgical operation and outcome

A bilateral frontal coronal hairline incision was given for resection and a one-stage operation for reconstructive surgery was performed on both patients, the mean operative time was 110 minutes.

#### 2.3.1 Case one

**Figure 5:**
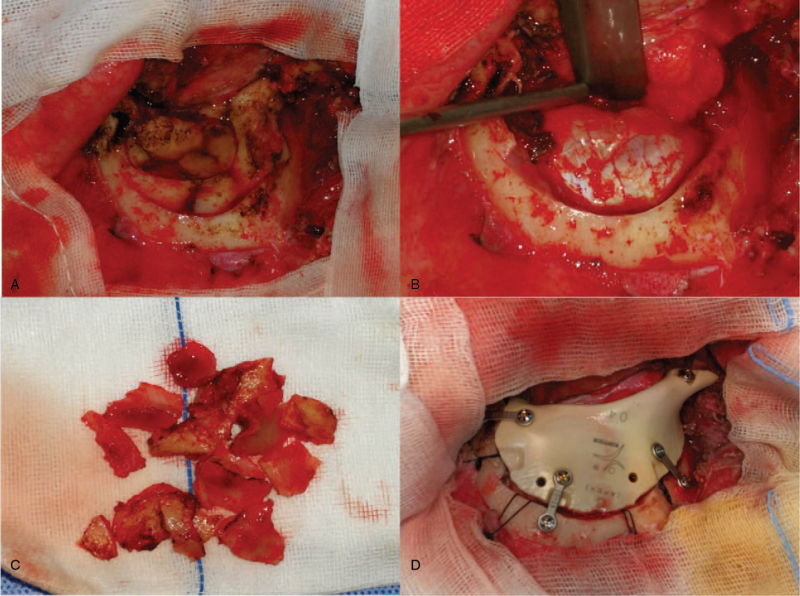
Intraoperative demonstration of case 1: A–C, exploration of the wound and removal of the crushed bone; D, implantation of the PEEK prosthesis with miniplates and screws.

After cleaning all the crushed bones as determined in the virtual surgical planning of the first patient, the operation field was repeatedly rinsed and disinfected with hydrogen peroxide and the frontal sinus was closed with thigh fascia. The customized implant was then well-matched with 4 straight locking mini-titanium plates in the defect area, and the crew holes were chosen to avoid frontal sinus (Fig. 5A–D). Antibacterial treatment was prescribed routinely for exposure of frontal sinus and prosthesis implantation. At a 32-month follow-up, the patient showed significant improvement in oculomotor deficits and regained a good appearance without any neurological deficits (Fig. 7A–D).

#### 2.3.2 Case two

**Figure 6:**
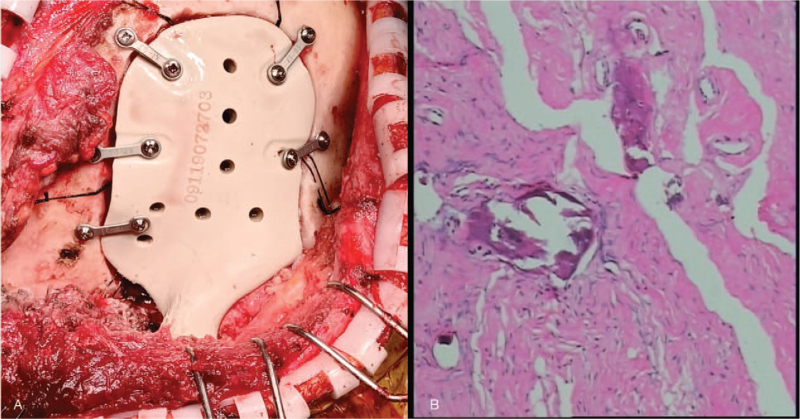
Intraoperative demonstration and postoperative pathology of case 2: A, implantation of the PEEK prosthesis with miniplates and screws; B, postoperative pathological examination revealed an osteoma with fibromatous hyperplasia.

Based on virtual surgical guilds and conducted by navigation's pointer in the second patient, the tumor resection margin was easily determined using a surgical sterile maker intraoperatively. After final verification of the resection borders, the neurosurgeon and ophthalmologist performed an overall resection of the neoplastic lesion, which included the right lateral anterior skull base and 1/2 of the right superior orbital zygoma. The edges of the defect were fully conformed to the PEEK implant fixed with 5 straight-locking mini-titanium plates (Fig. 6A). The postoperative pathological findings demonstrated an osteoma with fibromatous hyperplasia (Fig. 6B). The patient had an excellent restoration of contour and esthetics, as well as recuperation of orbital volume and ocular movement, without any neurological deficit, and the postoperative radiological examination demonstrated no evidence of tumor recurrence at a follow-up of 12 months (Fig. 8A–D).

**Figure 7:**
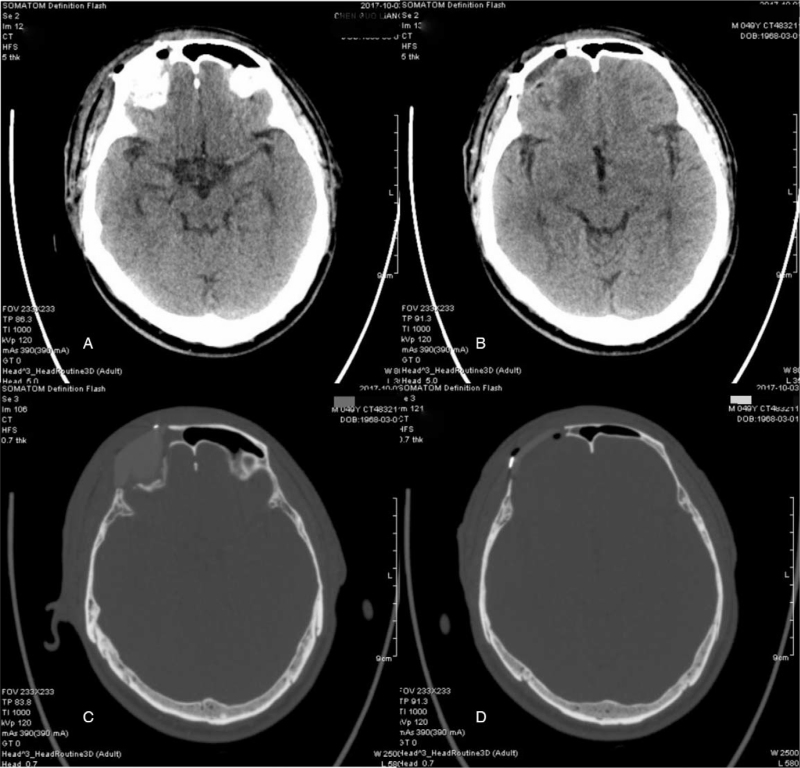
Postoperative CT scan showed a considerable degree of symmetry with PEEK prosthesis in case 1.

**Figure 8:**
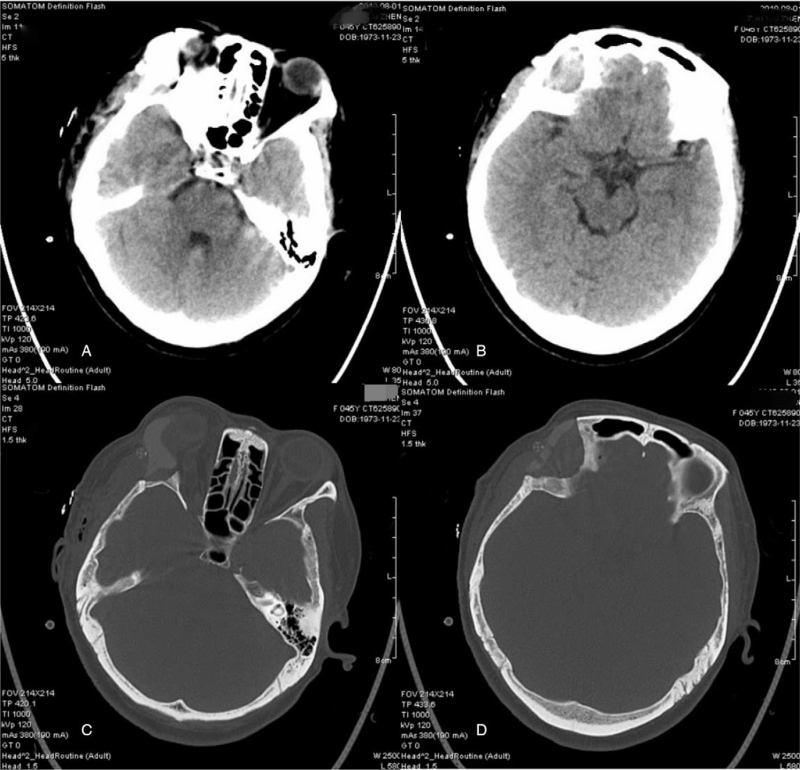
Postoperative CT scan showed a considerable degree of symmetry with PEEK prosthesis in case 2.

## 3 Discussion

Reconstruction of complex fronto-orbital cranial defects remains a surgical challenge because of its complex anatomy and postoperative complications.^[4,5]^ The therapeutic goal is to achieve not only cosmetic repair but also functional restoration.^[1,5,6]^ To that end, restoring the correct position of the globe and preventing postoperative restriction of the extraocular muscles is an approach that must be considered in this type of reconstructive surgery.^[1,9,10]^

Traditionally, autologous bone grafting remains a good reconstructive option for small single defects with irreplaceable histocompatibility, However, in the forehead-midfacial region, larger reconstructions not only lead to increased donor site morbidity and bone resorption, but also reduce the strength and plasticity of the aesthetic contour.^[2,5–9,11]^ TM and polymethyl methacrylate can provide good morphological results with computer assisted procedures, but nevertheless, preoperative or intraoperative bending and correction, and sometimes even excision to adjust the shape of periorbital ridges, especially in the orbit, is time-consuming and still difficult to accomplish.^[5,12,13]^ The correct placement of the implant and the conformity of its size and shape to the individual anatomy of the damaged structure are critical to the overall success of fronto-orbital reconstruction.

PEEK has become a favorable alternative for craniofacial reconstruction attributing to its perfect histocompatibility, excellent deformability, thermoplastic and ideal radiographic features since firstly reported by Scolozzi in 2007^[14]^ in a patient with gunshot fronto-temporal defect that had failed reconstruction with TM and methylmethacrylate.^[2–4,8,9,11,13]^ CAD–CAM is an emerging technology that has been used in recent years for cranioplasty of various materials.^[1,3,14,15]^ In this study, we innovatively used this technique, combined with virtual surgical planning and intraoperative navigation, to perform 2 reconstructions of cranial defects of different etiologies in the fronto-orbital region using PEEK implants. In our experience, CAD–CAM technology can provide a considerable degree of consistency in designing a mirror reconstruction by referencing the normal contralateral contour. As a result, all these customized patient-specific implants can be easily embedded, reducing surgical time and infection, while the technique is simpler and the aesthetic results are more satisfactory, making it possible to achieve individualized reconstruction in a single stage of surgery. For tumor patients, empirically, we recommend that the amount of bone to be resected should be generous in the virtual surgical planning phase in order to avoid an excessively time-consuming procedure of bridging the gap between the implant and the bone resection margin in case of underestimation of bone infiltration, regardless of the use of an intraoperative navigation system.

Generally, a critical factor in the pursuit of reliable and stable long-term results is the sterility assurance of implantation surroundings. In our experience, complex defects of the fronto-orbital skull are usually accompanied by extensive communication of the cranial cavity with the paranasal sinuses, and the frontal sinus should either be demarcated from the lesions area or be subjected to preliminary cranialization or obliteration. To ensure reliable frontal basal sealing and demarcation of the frontal sinus bone defect, the use of a microvascular free flap is highly recommended.

## 4 Conclusion

We presented 2 rare cases of fronto-orbital cranial defects under virtual surgical planning guidance with CAD–CAM patient-specific PEEK prostheses for one-stage reconstructive surgery. An emphatic intraoperative accessibility of the customized PEEK prosthesis and excellent level of aesthetic satisfaction of patients making this manner an optimal alternative for this particular subtype craniofacial defect.

## Author contributions

**Conceptualization:** Min Yang.

**Data curation:** Min Yang.

**Resources:** Zhangyi Wu, Hai Yu, Jun Cheng.

**Writing – original draft:** Min Yang.

**Writing – review & editing:** Hai Yu, Jun Cheng.
